# How to Select the Proper Potency

**Published:** 1890-03

**Authors:** 


### HOW TO SELECT THE PROPER POTENCY.

The following instructive and interesting discussion is extracted from the proceedings of the International Hahnemannian Association, and was started by a paper of Dr. B. Fincke, entitled "Contributions to Materia Medica." We have chosen it knowing that it will profoundly *interest the majority of our readers.* [EDS.]

DISCUSSION.—Dr. Emory—I have listened to that paper with deep interest; it is right along the line of thought which have exercised me considerably and often. Dr. Fincke has certainly shown some light which has left us in as bad a mess as we were before. I would like to know if Dr. Fincke has found out how we are to individualize our cases in regard to potencies in each case. Is there any other way except empiricism? Are there any instructions to select the different potencies for different patients? I am sure homœopathicians all over the world would be thankful to Dr. Fincke for some light on this subject.

Dr. Reed—It seems like something wonderful about the action of potencies on the human organization. I have a case in mind of a patient sick unto death from malarial fever and treated by an allopathic physician on account of the distance from a homœopath. I was afterward requested to make a visit. The simillimum was Sulphur. I gave a potency 51 M, Fincke, and sometimes CM, whenever necessary to go higher. The man was cured with one dose. In four weeks afterward he had prosopalgia of right side of face occupying whole of the right side—with extremely acute, lancinating, cutting pains aggravated by heat—thermometer 18° below zero out-doors—and when near the stove the pains were worse, but when cold the pains were easier, could not bear the heat.

There is only one remedy, Bryonia 2 C. I gave him two or three powders to take with him. He went home and took the powders four hours apart and came back no better. I gave him another dose of the same potency and he went home and took it and came back no better and much discouraged. "I will cure you right away," I said, and I went to my case and took a bottle of Fincke's 76 M and gave him a dose on his tongue and in five minutes he said, "Doctor, that is my medicine." Why did Bryonia 2 C do no good? Because he had had a potency of Sulphur 51 M and he had become used to high potencies and a low potency could not act where a high potency had been acting.

It was the same way with a cough which Professor Kent had been treating. I said Phosphorus (was the remedy) and the question was—as she received no benefit—why was it? Because she had always received Phosphorus CM, and Phosphorus 2 C, did her no good, but the CM potency cured her in five minutes.

Dr. Butler—It is all true, but how to do it is the question. My father had a brother five feet seven inches high, and how is it they were not the same height? We don't know. I am quite agreed to admit the same need to make the remark you will all accept in the very line Dr. Emory did. These are all well, and the paper is valuable as to the similitude of the dose as well as the similitude of the drug; but at present we have no knowledge how to choose the potency of the remedy, but may give experiments. I believe that the lower potencies are more safe for action in acute cases, and the higher in chronic cases; perhaps because I have a tendency to repeat in acute cases pretty quickly. I am going to use a tincture of 2 C for acute cases and try it, and tell you by and by.

Dr. Reed—They can be safely repeated provided you don't repeat too far. Give the remedy in solution, and watch the patient until you get a satisfactory action of the drug; then stop, and you are safe.

Dr. Schmitt—I have taken that subject into consideration many times, because I have had cases where only certain potencies would act. I remember the case of a man who was a continual drinker, and his stomach was out of order very often. He came once to me with colic in the stomach caused by drinking, and I gave him Nux-vom.$^{2c}$, and he had hardly taken it, and while I was making up some more powders, when he gave one eructation and said: "I am all right"—all the pain was gone. I gave him a few powders to take when the pain came on again. It lasted half an hour or longer. He came to me again and wanted some powders. I knew I had given him Nux-vom. (I did not take the case down), but I had forgotten the potency, and as I generally prescribe the CM, I gave him the CM. He went home and came back in the evening, and said: "the powders did not do any good." He took them on a full stomach. I then remembered I had given him Nux-vom.$^{2c}$ in the first instance. I gave him this accordingly, and he hardly had it on his tongue before he was all right. He went into other hands for an attack of pleurisy because I wanted to force him to high potencies. I did not know as much as I do to-day. I gave during the attack of pleurisy Aconite$^{cm}$ and Bryonia$^{cm}$, but did not get a bit of response. Then he called in another physician. I should have given that man 2 C, and I would have then got a response from the remedies.

Dr. Reed—You established a precedent in your 2 C potency.

Dr. Schmitt—That may be, although I have sent out a kind of feeler, as Sulphur or Sepia, especially given in the 3 and X, then the 2 C, CM, and MM, especially in cases of consumption where Sulphur is indicated. I would rather give 2 C of Sulphur first instead of the CM.

Dr. E. T. Adams—I have long known how difficult it was in my own cases to find the indicated remedy, but I begin to feel from the present discussion that it is much more difficult to find the indicated potency.

Dr. Emory—Dr. Reed said that Dr. Schmitt had established a precedent in the first prescription of 2 C. Is there anything in that? Can you establish a precedent of that kind? It is contrary to my experience in the treatment of chronic cases. I nearly always begin with the 2 C, and let it act until it ceases. Then if there is no change of symptoms, I give a higher, and find the higher potencies act more efficiently in these cases. A precedent was not established by the 2 C. I generally give a higher potency if the remedy is well indicated.

Dr. Reed—I don't know if that is true. Here is a case that occurred after confinement. I knew her remedy was Calc-carb. I had given Calc-carb. before her confinement (2 C), and I thought to myself, I will give Fincke's 85 M. I gave her 85 M of Calc-carb., but no benefit accrued therefrom. All the symptoms remained the same. I did not know what to do, and hesitated for two or three days and then gave *Calc-carb.*, 2 C, and she rested well afterward. I cannot explain this thing to Dr. Emory. It will have to be explained in the future.

Dr. Custis—This is the most important discussion started since the meeting convened, and it opens a subject which has shown just what this Association is in existence for, and I have studied it more than any other one. Dr. Butler will miss it if he divides his cases into acute and chronic. Some people are made for one potency, and some for another, perhaps, but the difference is in the nature of the disease. Diseases which depend upon change of function, such as Dr. Schmitt's man, can be met with a lower potency with effect; but if there is organic change, for that I would much prefer, and think better results come from higher potencies, and if the acute condition is ingrafted upon the already organically diseased organ, the higher and highest act the quickest, but will not bear any repetition at all.

If Dr. Schmitt's patient had an organically diseased stomach, and then had gone on a spree, he would have had a better result in the higher potency than in the 2 C. It depends more on the constitutional condition of the patient, and whether the disease is purely functional or threatens organic changes.

High potencies are more prompt, and the only ones that will cure diseases where there is organic change such as tubercular meningitis, and then they must be used carefully and not repeated.

Dr. Schmitt—Is pleurisy an organic change?

Dr. Custis—It is after it has started—that depends on your man. In some cases a low potency man cannot be acted upon by high potencies. Sometimes I get patients who have been all around before they could get any results from medicines. It seems to me to be more in the peculiarity of the patient.

Dr. Biegler—This field is an unexplored one to me, and I have never been able to obtain a guide by means of chart and compass to steer myself with, and the only point in my mind in this discussion on which I am quite satisfied is that in acute diseases, such as diphtheria. In that disease I have never cured a case with low potencies, and I think if I get a case in time and no interference, I never lose a case of diphtheria with the high potencies. Also my experience is that I seldom have to repeat the dose. They recover on the single dose in the majority of cases. I found that in a very severe case where the remedy (Belladonna) cured in four or five days with a single dose. When we say that we ought to prescribe the high potencies in chronic cases, I am doubtful. Here is an illustration that invalidates that proposition—how can I give you any light except I select the potency according to the susceptibility and sensitiveness of the organism of the patient?—I may say, "*by guess work.*"

Dr. Emory—I don't know whether it would meet with favor or not. I move that we request Dr. Fincke to follow up this paper with any light he can throw upon it.

Dr. Biegler—I wish to suggest that a vote of thanks be given Dr. Fincke, and an invitation to continue his good work and to become a member of this Society (and pay his fee).

*Motion* (President)—It is moved and seconded that a vote of thanks be tendered to Dr. Fincke for the able paper he has here presented, and an invitation or request be extended to him that he may at a coming meeting also present his further views in regard to his observations as to the result of the remedies and their potencies. Carried.

Dr. Sawyer—We have had during the past fall and winter in our town an epidemic of diphtheria. Under the allopathic treatment whole families died. We have had an exceedingly bad type of diphtheria there—some children died inside of twelve hours, in spasms, from the time of taking the disease. I had my full share of cases, and in no instance did I give any potency below the 5 M. I lost no cases and rarely repeated my remedy. I also had a case of acute interstitial nephritis with black urine and swelling of the body from head to foot. Lachesis was the remedy given in the CM potency with slight amendment; then I gave one dose of the 11 MM (Fincke), and in twenty-four hours the patient was cured and the black urine gone.

Dr. Kimball—Are you sure they were cases of diphtheria and not follicular tonsillitis?

Dr. Sawyer—The best physicians and surgeons said it was true diphtheria. It was something that killed in a few hours under allopathic treatment.

Dr. Stowe—However much we may search for the square rule for the selection of the potency, we shall never reach any other safe guide than that which the lamp of experience gives us. Judgment and experience must be the only guide by which to select the remedy and potency.

Dr. Long—The remedy Calc-carb. was mentioned. Has any physician had a quick and prompt action from Calc-carb. in chronic cases? Dr. Reed waited two or three days; I waited six weeks when I gave any of the CM potency.

