# Corrections

**Published:** 1890-04

**Authors:** 


CORRECTIONS.—March number, page 119, sixth line from the bottom, for organization read *organism*. Same page, second line from bottom, for 51 M read 55 M. Page 120, thirteenth line from top, for 76 M read 70 M. Page 120, sixteenth line, for 51 M read 55 M.

